# A Retrospective Analysis of Patients with Short Stature in the South of China between 2007 and 2015

**DOI:** 10.1155/2018/5732694

**Published:** 2018-12-20

**Authors:** Su Wu, Qian-qi Liu, Wei Gu, Shi-ning Ni, Xing Shi, Zi-yang Zhu

**Affiliations:** Department of Endocrinology, Children's Hospital of Nanjing Medical University, Nanjing 210000, China

## Abstract

**Objective:**

To describe the demographic features of children with short stature and poor growth in the south of China and provide better guidance on clinical strategy and decisions.

**Study Design:**

This retrospective, chart review study analyzed children with short stature and poor growth admitted to the Department of Endocrinology of Children's Hospital of Nanjing Medical University from Jan 2007 to Dec 2015.

**Results:**

The chart review yielded 4142 patients, including 2546 boys and 1596 girls (*P* < 0.001); the number of patients gradually increased per year from 2007 to 2015. There was an upward trend in the average levels of height standard deviations (SDs) during the study period (*P* < 0.001), both in males (*P* < 0.001) and females (*P* < 0.001). Mean height SDs were smaller in females (-2.42±1.09) than males (-2.33±1.03;* P* = 0.01). The percentage of females admitted at normal height (33.83%) was lower than that of males (37.20%;* P* = 0.028). The peak age range of hospitalization in males was 10–12 years of age, while females were generally admitted earlier—8–10 years.

**Conclusions:**

There was an increasing tendency to focus on children's height. Parents and pediatricians were recommended to pay more attention to the treatment needs of girls while avoiding excessive treatment of those who merely appear not to be tall enough without a clear medical issue related to growth, especially for boys.

## 1. Introduction

Short stature is a diagnosis given to approximately 2% of individuals and refers to a height that is over two standard deviations (SDs) below the average height for age, gender, and race [1]. Abnormal growth may suggest the potential diseases (e.g., GH deficiency [GHD], gestational age [SGA], Turner's syndrome, etc.) in a normal-appearing child. Historically, people have believed that tall men are more likely to be successful [2]. Physical height has been associated with self-esteem [3, 4] and children with short stature experience impairments that impact their academic performance and social life [3–7]. In contrast, it has also been suggested that short stature has no significant effect on normal psychological development [8–10], or only conferred minor difficulties in the behaviors of boys with short stature [11], without leading to negative behavioral outcomes and interpersonal relationships [12]. Although the evidence that short stature is associated with psychosocial stress is lacking, many children (including those who later proved to have no health issues) have visited pediatric endocrinology clinics due to short stature [13, 14]. Previous research has shown that males (boys) have been the preponderant gender visiting doctors for growth hormone (GH) treatment [15–21], although girls' growth showed greater impairment in terms of a greater proportional reduction in final heights [14, 22]. It is common knowledge in China that men are considered socially more important than women by the culture at large, but there is limited information regarding the treatment strategy for short stature specific to age and gender.

This study describes the clinical data of children admitted to the endocrinology department of Children's Hospital of Nanjing Medical University for the evaluation of short stature and poor growth during the period of 2007–2015 in the south of China. Our study focuses on the age, gender, height, and distribution characteristics for China and other countries with respect to slow growth in children and treatment, to provide better guidance in clinical strategy and decisions.

## 2. Subjects and Methods

### 2.1. Subjects

Our study was approved by the Ethics Committee of the Children's Hospital of Nanjing Medical University, China. Informed consent was obtained in written form all patients.

All the subjects in this study were patients previously enrolled from chart reviews based on the database of Children's Hospital of Nanjing Medical University; specifically, data were collected from all children (N = 4284) referred to our ward for short stature or poor growth between January 2007 and December 2015. Our study only included new cases complaining of poor growth or short stature. Patients without data from the initial evaluations of height and weight were excluded (n = 35), as were patients without a final diagnosis (n = 107). Among the remaining nonexcluded patients (n = 4142), the patients were aged 1 to 18, with an average age of 8.91 (± 2.95) years. The heights of 2655 patients were less than or equal to -2 standard deviations (SDs) SDs (-11.33 SDs to -2 SDs), which was diagnosed as “short stature”; 1487 were greater than -2 SDs (-1.99 SDs to 0.93 SDs) and fell under the category of “normal height” (even if on the short side of that spectrum).

A comprehensive set of diagnostic tests was performed, including a GH stimulation test, IGF1, bone age, biochemical and blood tests, and other related tests. Subsequent to these tests, 2278 patients were found to have an organic disease potentially related to compromised growth: 2014 had GH deficiency (GHD), 32 were small for gestational age (SGA), 65 had chromosomal disease, 58 had short stature with a systemic disease (congenital heart disease, chronic renal failure, renal tubule acidosis, Bartter syndrome, Gitelman syndrome, diabetes insipidus, Duchenne and Becker muscular dystrophy, or intracranial tumor), and 66 had multiple pituitary hormone deficiencies. Other conditions represented were hypothyroidism, skeletal development disorder, and Intracranial tumor. 1715 had idiopathic short stature (ISS), 111 were cases of familial short stature (FSS), and 38 were cases of delayed puberty. An extensive list of the various pathologies with the number is shown in Table 1.

GH exercise challenge test: the children sat with an empty stomach for more than 15 min, and the GH level was measured; then, they walked for 15 min and ran for 5 min, making their heart rate post exercise more than 120 beats/min, and the GH level was tested again. The GH exercise challenge tests of all children were initially improved in the outpatient clinic before admission. The patients with a GH exercise secondary value > 10 ng/ml were excluded from GHD and treated in the outpatient clinic; patients with a GH exercise excitation value < 10 ng/ml were admitted to hospital for further examinations.

GHD definition: the GH peak of GH stimulation test is less than 10 ng/ml, the blood IGF-1 level is low, the bone age is two years younger than chronological age, the annual growth rate is < 2 SDs for chronological age, and CT or MRI examination has excluded an intracranial tumor [23]. Among them, a GH peak < 5 ng/ml, a diagnosis of complete GHD; a GH peak 5–10 ng/ml, and a diagnosis of partial GHD.

Standards for receiving GH treatment: Refer to the guidelines for the diagnosis and treatment of children with short stature [24], including the short stature caused by GHD, ISS, SGA, Turner syndrome, and chronic renal failure.

### 2.2. Methods

Basic information including patients' gender, age, bone age, height, weight, final diagnosis, and laboratory and radiologic evaluations was collected from electronic medical records. Height, weight, and pubertal status were assessed and documented by a pediatric endocrinologist. In particular, patients' height was derived from the average value of three continuous measurements and expressed in SDs using height-standardized charts for Chinese young children aged 0–18 [25].

### 2.3. Statistical Analysis

The statistical analysis was performed using SPSS 19.0 software (IBM, Armonk, New York, USA). The results were described as the mean ± SD unless otherwise stated. Differences between means in boys and girls were evaluated using a* t*-test. Differences between multiple groups were analyzed using analysis of variance (ANOVA) and then least significant difference (LSD) for continuous variables. Data in skewed distribution were analyzed by Kruskal-Wallis test and then all pairwise multiple comparisons. Comparisons between the constituent ratios were done using the chi-square (*χ*^2^) test.* P*-values less than 0.05 were considered statistically significant.

## 3. Results

**Figure 1:**
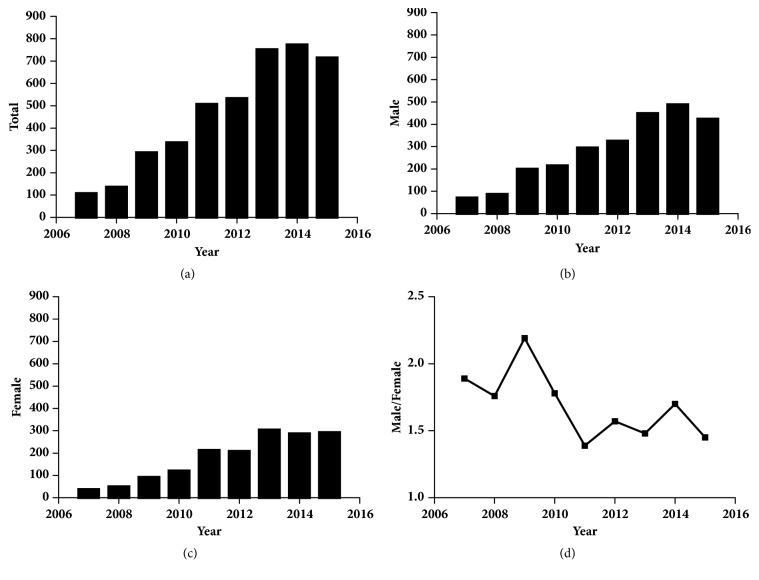
Number of patients for the entire group (a), males (b), and females (c) admitted each year. (d) male: female ratios from 2007 to 2015.

Patients' basic information (n = 4142), including age, bone age, gender, height, and weight and height SDs at presentation, is shown in Table 2. Annually, between 107 and 773 patients (median, 472) were treated at the clinic, and the number of patients increased gradually from 2007 to 2015 (Figures 1(a), 1(b), and 1(c)). Among the 4142 patients, the male-to-female ratio was 1.6:1 (2546 boys and 1596 girls; T = 183.176,* P* < 0.001). The distributions of male-to-female ratios by year are shown in Figure 1(d), which remained relatively stable over the last nine years (*χ*^2^= 13.965,* P* = 0.083).

**Figure 2:**
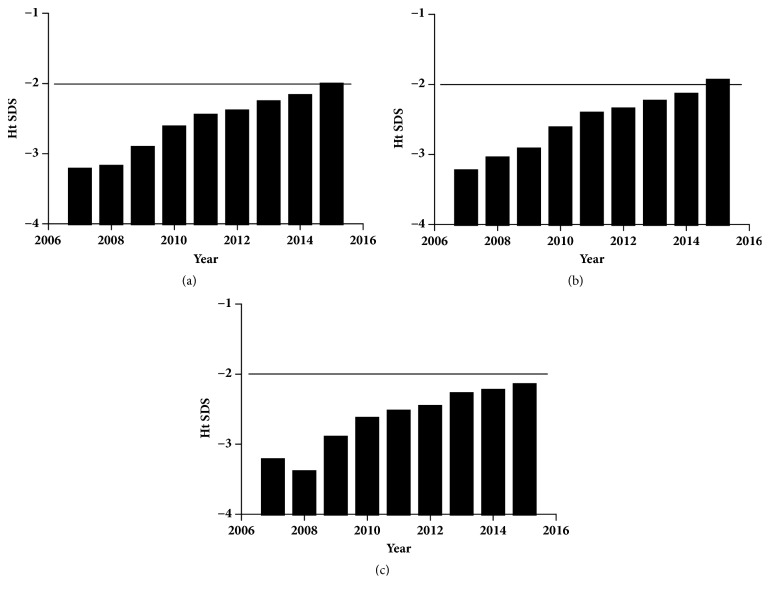
Histogram of height* z* scores at the time of the first visit. Height SDs for the whole group (a), males (b), and females (c) sorting by years. The average level of height SDs of males exceeded -2SDs in 2015.

The distribution of height SDs over the whole period is shown in Figure 2. An upward trend was present over time in the average level of height SDs (F = 50.899,* P* < 0.001, Figure 2(a)), both in males (F = 38.556,* P* < 0.001, Figure 2(b)) and females (F = 15.243,* P* < 0.001, Figure 2(c)), especially for males. It was noteworthy that the average levels of height SDs exceeded -2 SDs in 2015 for males. For the general population, females were shorter in the mean height SDs (-2.42±1.09) than males (-2.33±1.03; F = 6.695,* P* = 0.01).

**Figure 3:**
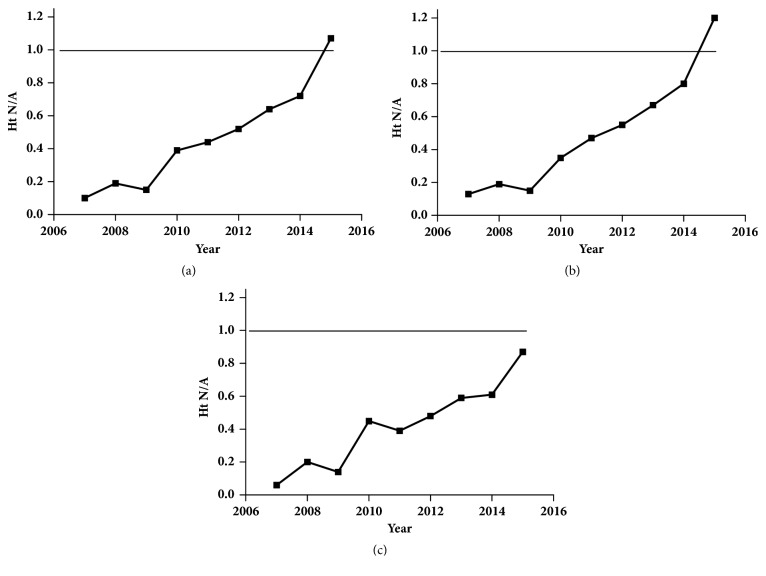
Normal-to-abnormal height ratios for the whole group (a), males (b), and females (c) sorting by years. The normal: abnormal height ratio of males exceeded 1 in 2015.

The percentage of the whole group admitted at normal height was 35.90%. The proportion of boys admitted at normal height (37.20%) was significantly higher than females (33.83%) (*χ*^2^ = 4.816,* P* = 0.028). The distributions of normal-to-abnormal height ratios every year are shown in Figure 3; the normal-to-abnormal height ratios increased year by year (*χ*^2^ = 235.915,* P* < 0.001, Figure 3(a)), both in males (*χ*^2^ = 175.148,* P* < 0.001, Figure 3(b)) and females (*χ*^2^ = 66.746,* P* < 0.001, Figure 3(c)), with a more pronounced increase in males. Of note, the normal-to-abnormal height ratio of males exceeded 1 in 2015. The percentage of girls (54.20%) with an organic disease was comparable to boys (55.50%) (*χ*^2^= 0.195,* P* = 0.659).

**Figure 4:**
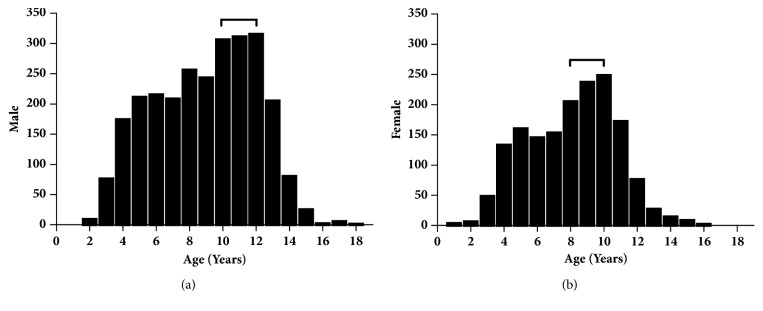
Histogram of males (a) and females (b) by ages (years). The peak age for hospitalization was 10–12 years in males, 8–10 years in females.

For the entire group of patients, the average age of admission was 8.91 ± 2.95 years. Males were older than females, with the average age of females being 8.39 ± 2.66 years and males 9.24 ± 3.10 years (F = 63.284,* P* < 0.001). For average bone age, males (6.88±3.13 years) were younger than females (7.04 ± 2.90 years; F = 12.702,* P* < 0.001). The distributions of males and females by age are shown in Figure 4, demonstrating that the peak age range of admission to the hospital was 10–12 years in males and 8–10 years in females.

Of the 4142 patients, 1403 received GH treatment, including 865 boys and 538 girls, with a male-to-female ratio of 1.61.

## 4. Discussion

The number of children with short stature has increased on a yearly basis in the south of China during the last nine years. The average level of height SDs and normal-to-abnormal height ratios of these patients have increased each year, indicating that the focus on children's height has increased over the past nine years. Despite the absence of evidence supporting the hypothesis that short stature is closely related to psychosocial stress, psychological adaptation, or academic achievement [26], most parents would bring their children to the hospital for the diagnosis of short stature [26, 27]. Moreover, other potential factors are related to referral patterns, such as the development of society and economy, genetic factors, or the socioeconomic and cultural background of parents.

Our number is based on patients referred to the endocrine ward by pediatric endocrinologists. Although the normal-to-abnormal height ratios increased on a yearly basis from 2007 to 2015 in our study, the ratio in this study (34.68%) was lower than that in other studies (89.4% in Korea [13] and 63.7% in the United States [14]). Although there are many potential explanations for the results, such as the difference in referral criteria or social factors between countries, our findings suggest that the growing problems of children in the south of China are more severe than those in Korea or the United States.

Our findings showed that the age of hospitalization could play a role in hospitalizations for the evaluation of poor growth. The peak age of children is mainly in the prepubertal years (10–12 years for boys and 8–10 years for girls). Early diagnosis and treatment are good for height improvement in patients with GHD and Turner's syndrome [28, 29]. Thomas [30] noted that children with short stature are usually not diagnosed in a timely manner, leading to the loss of the chance to improve height outcomes. In our study, all patients were admitted at a relatively late age, adding to the difficulty of treatment. For early detection, diagnosis, and treatment, multiple measures should be taken at different levels. On a social level, the government and other social organizations should strengthen education and create awareness campaigns to improve the knowledge of short stature. First, government publicity (television, internet, and paper media) on short stature should be strengthened. Second, the development and implementation of child health work should be strengthened. Third, the government's medical insurance for short stature treatment should be strengthened to allow more children to obtain medical treatment. Fourth, it is important for school to carry out regular physical examinations every school year. Lastly, community hospitals should strengthen community doctors' understanding of short stature and promptly refer children with growth problems to the hospital. At the hospital level, pediatricians should be able to diagnose the children with short stature timely, especially for the children with feeding difficulties, low birth weight, and chronic diseases and congenital diseases, as well as premature infants. On a family level, parents should send their children to the hospital in time when growth problems surface. Taken together, early diagnosis and treatment of short stature will lead to a better curative effect.

Our study also demonstrated that gender played a role in hospitalizations for the evaluation of poor growth. The male-to-female ratio for the entire group was 1.60, which was lower compared to previous studies [14–18]; there was a slightly descending trend for the male-to-female ratios during the last nine years. The lower ratio might be related to the referral pattern, racial/ethnic or cultural differences, and so on. Although males outnumbered females, our results revealed an interesting contrast: girls were shorter than boys. Grimberg found that girls' heights showed more impaired growth, with a median height of -2.4 SDs for girls and -1.9 SDs for boys [14]. Besides, the normal-to-abnormal height ratio of girls was lower than that of boys, consistent with the results of previous studies. Taken together, these findings provided a fuller picture of the gender difference. Gender distribution was also related to age at the initial diagnosis. Males were older than females upon initial examination, due to puberty starting later in boys. Boys who are shorter than their peers during the sexual development phase of their lives are considered as potentially having growth-related health issues. However, boys might continue to grow when epiphyses have been fused in most girls. Gender differences also exist in the GH treatment recommendations of experts. In our study, we treated patients according to published management guidelines for children with short stature [24]. Among 4142 patients, 1403 received GH treatment, including 865 boys and 538 girls, with a male-to-female ratio of 1.61, consistent with a previous study [31]. Of the 1403 children treated with GH, only 299 had complete GHD. GH registries indicate the preferential treatment of boys with short stature. The pediatric endocrinologists suggested GH therapy for male patients 1.3 times as often as female patients [31]. In countries with the greatest GH use, boys underwent GH treatment by the ratio of about 2:1 compared to girls [18–20].

Gender discrepancies (especially in terms of the treatment degree) have existed for the last nine years in our hospital. Provided that short stature is a kind of physical disability, girls with poor growth should be treated the same as boys. It is unclear what has caused such gender discrepancies; however, patients, families, doctors, and social factors may account for them. Since social pressures are greater for boys than girls in terms of height, boys would prefer to receive specialist care. This may be especially true (and problematic) in China, as the view that men are superior to women is quite normal in China.

ISS children receiving medical evaluation are asked more psychosocial questions than normal children or ISS children without medical evaluation [32]. This fact partly indicates the worrisome consequences of the overtreatment of boys. Further, more serious long-term issues, including late diagnosis and treatment, should be addressed. It is noteworthy that the proportion of patients with GHD (2014 out of 2278) is higher in this paper. As all the children included in this study were short children admitted due to the GH challenge test, the proportion of GHD in this study is higher.

## 5. Limitations

There are several limitations of this study. First, this article is a retrospective study. Although the number of visits of children with slow or short growth is increasing each year, and the ratio of males to females is decreasing, the related reasons were not studied. Second, we failed to study the underlying causes of gender differences in treatment for short and slow-growing children. Finally, although there are gender differences in the treatment of short stature, data on whether there is a gender difference in treatment benefit have not been obtained, and further research is needed.

## 6. Conclusions

There was an increasing tendency to focus on children's height during 2007–2015 in the south of China. To summarize, the present findings indicate that age and gender played roles in hospitalizations for the evaluation of poor growth. The females (8–10 years) were generally admitted earlier than males (10–12 years). There were several gender discrepancies, including the male predominance in patient quantity and GH treatment and female disadvantages in height. However, these findings were not restricted to children with short stature in the south of China. In 2017, a retrospective study of prepubertal children with short stature enrolled from Europe, the United States, and Japan also demonstrated the male predominance in patient quantity and GH treatment as well younger and shorter females compared with males [33].

In conclusion, male predominance is a global phenomenon. We suggested that society at large, parents, and pediatricians should pay more attention to girls with poor growth, avoiding missed or late diagnosis of the disorder. Meanwhile, excessive examination and treatment for children who do not appear tall enough should be avoided. Therefore, firm evidence supporting the performance of treatments is needed, especially for boys.

## Figures and Tables

**Table 1:** Etiology classification of 4142 patients with short stature.

**Etiology classification**	**Cases**	**Boy/girl**

**Short with disease**	**2278**	**1413/865**

GHD	2014	1293/721

Complete GHD	627	431/196

Partial GHD	1387	862/525

MPHD	66	52/14

Hypothyroidism	9	2/7

SGA	32	18/14

Skeletal development disorder	19	10/9

Intracranial tumor	15	9/6

Chromosomal disease	65	3/62

Turner syndrome	62	0/62

Other chromosomal disease	3	3/0

Chronic systemic disease	58	26/32

**Short without disease**	**1864**	**1133/731**

ISS	1715	1030/685

FSS	111	71/40

Physical puberty delay	38	32/6

Total	4142	2546/1596

GHD, growth hormone deficiency; MPHD, male patients with heroin dependence; SGA, small for gestational age; ISS, idiopathic short stature; FSS, familial short stature.

**Table 2:** Basic information on patients.

**Year**	**Cases**	**Age** **(year)**	**Bone Age** **(year)**	**Male/** **Female**	**Height** **(cm)**	**Weight** **(kg)**	**Ht SDS**
2007	107	8.97±3.23	6.38±2.82	70/37	115.73±15.61	21.74±7.22	-3.22±1.18

2008	135	9.51±3.09	6.95±3.02	86/49	118.36±14.91	23.48±8.37	-3.18±1.28

2009	290	9.92±3.00	7.20±2.93	199/91	122.21±14.69	24.74±7.89	-2.91±1.12

2010	334	9.26±3.06	7.12±3.20	214/120	120.71±16.36	24.64±8.83	-2.62±1.29

2011	506	8.78±2.96	6.75±2.95	294/212	119.07±15.17	23.30±7.18	-2.46±0.98

2012	532	8.85±3.06	6.84±3.23	325/207	120.57±15.92	23.94±8.21	-2.39±0.92

2013	751	8.86±2.84	6.93±2.98	448/303	120.58±15.11	24.06±7.83	-2.26±0.95

2014	773	8.58±2.87	6.97±3.00	487/286	120.58±15.38	24.58±8.46	-2.18±0.88

2015	714	8.74±2.82	6.99±3.08	423/291	120.54±14.94	23.86±8.41	-2.01±0.98

Total	4142	8.91±2.95	6.94±3.04	2546/1596	120.20±15.35	24.03±8.13	-2.37±1.05

## Data Availability

The datasets supporting the conclusions of this article were included within the article.
